# Metabolically Healthy Obesity (MHO)—New Research Directions for Personalised Medicine in Cardiovascular Prevention

**DOI:** 10.1007/s11906-020-1027-7

**Published:** 2020-02-17

**Authors:** Peter M. Nilsson, Johan Korduner, Martin Magnusson

**Affiliations:** 1 Department of Clinical Sciences, Department of Internal Medicine, Skåne University Hospital, Lund University, Jan Waldenströms gata 15, 5th floor, S-20502 Malmö, Sweden; 2 Department of Cardiology, Clinical Research Centre (CRC), Jan Waldenströms gata 35, S-20502 Malmö, Sweden; 3 Wallenberg Centre for Molecular Medicine, Lund University, Malmö, Sweden

**Keywords:** Cardiovascular, Diabetes, Health, Metabolic healthy obesity, Microbiome, Protection

## Abstract

**Purpose of Review:**

To discuss new findings on the heterogeneity of obesity and associated risks.

**Recent Findings:**

Obesity is a public health problem of immense importance on a global scale. However, epidemiological findings and clinical studies have revealed that obesity is a heterogeneous phenotype and that not all obese subjects run the same risk for complications. Current research has tried to describe so-called metabolically healthy obesity (MHO), defined by lack of risk factors included in the metabolic syndrome. These subjects will not escape long-term complications, but mortality risk is not increased. However, a new definition of MHO has recently been proposed, based on the lack of hospitalisation for somatic disease for decades in middle life. MHO subjects defined in this way are characterised by being “fat and fit” and also run a lower risk of long-term complications.

**Summary:**

If MHO could be better understood, this could contribute to a more diverse clinical approach to obesity based on personalised medicine.

## Introduction

Even if obesity is a well-known risk factor for many aspects of morbidity and mortality [1], as well as causing a high economic burden to society [2], not all obese subjects have the same increased risk, thus showing heterogeneity. In fact, there are examples indicating that obesity may be protective when chronic disease is already present, for example chronic obstructive pulmonary disease (COPD) or congestive heart failure (CHF), when the prognosis seems to be better in more overweight or obese subjects than in leaner ones [3]. This has been referred to as cardiac cachexia in patients with CHF, linked to a state of chronic inflammation that could impact on weight loss and the associated worse prognosis [4]. Even subjects without chronic disease in mid-life have a better prognosis if they stay weight-stable in mid-life as compared with weight increase or weight loss based on observational data [5].

Also in subjects without such chronic disease conditions the negative impact on health of overweight/obesity is not uniform and there are many examples in the clinical setting, or based on population studies, that not all obese subjects will develop for example elevated blood pressure or impaired glucose metabolism that would otherwise be expected.

## Does Metabolically Healthy Obesity (MHO) Exist or Not?

This finding of heterogeneity of risk has provoked a debate whether a phenotype of metabolically healthy obesity (MHO) exists or not, based on a definition of lacking features, or variables, of the so-called metabolic syndrome or by the level of insulin resistance, mainly by using the homeostasis model assessment of insulin resistance, HOMA-IR [6••, 7]. Protagonists of this hypothesis have shown data that such MHO individuals exist in epidemiological studies, but critics have stated that this represents only a temporary state and that sooner or later the burden of risk factors will become more visible and that the cardiometabolic event risk will therefore increase [8•]. Thus, according to this view, MHO is at best a temporary state and will not last, ultimately transforming into a metabolically unhealthy status [9]. Recent meta-analyses, based on this traditional definition of MHO, have summarised that indeed the long-term risk of cardiovascular disease (CVD) is increased in former MHO subjects, but not total mortality risk [10••]. A new updated meta-analysis on the potential role of MHO in modifying risk of cardiovascular disease, total cancer, and all-cause and cause-specific mortality is presently underway based on published studies up until December 2019 according to a published protocol [11].

Another disputed topic is what kind of reference group should be used to compare MHO subjects with for risk estimates and clinical characteristics. Should it be the general non-obese population or a more selected subgroup of metabolically healthy, non-obese subjects as well as normal-weight individuals with a metabolically healthy phenotype [12]? This question is still not settled. If a very healthy reference group is selected, most other groups will show increased risk, but if the general population is used as reference, the comparison more resembles the real-world experience.

## A New Definition of MHO Is Presented

To overcome these theoretical and conceptual problems, we have recently proposed a new definition to be used for classifying MHO subjects, based on data from two population-based studies in Malmö, southern Sweden. In the first study from the Malmö Preventive Project (MPP), we defined MHO subjects as the ones escaping hospitalisation for several decades of their mid-life and not developing type 2 diabetes, i.e. no transition to diabetes [13]. This group of non-hospitalised, non-diabetic MHO subjects with advanced obesity (BMI > 35 kg/m^2^) could well have a history of different risk factors or prescribed drugs, but the argument is that these traits or risk factors did not reach a point when need of hospital care was necessary, i.e. avoidance of hospitalisation. Thus, this MHO group could be compared in cross-sectional analyses with other similarly obese subjects with a history of hospitalisation as well as with non-obese controls. The findings indicated that the MHO group was characterised by a non-sedentary behaviour and therefore more like “fat and fit” than the obese group with a history of hospitalisation [13].

In a second study, based on the same definition of non-hospitalisation for decades in mid-life, data from the prospective Malmö Diet Cancer Study (MDCS) was used [14••]. It was found that MHO subjects (BMI ≥ 30kg/m^2^) without any hospitalisation for somatic health care before baseline in 1992 to 1996 (with exclusion of hospitalisation due to trauma or normal deliveries) had a significantly lower incident risk of prospective CVD and mortality as compared with Metabolically Unhealthy Obese (MUO) subjects (e.g. non-diabetic subjects with BMI > 30 kg/m^2^ that had been hospitalised). In fact, the prospective risk of MHO subjects was similar to the one of non-obese controls, both metabolically healthy and unhealthy non-obese subjects [14••]. We are now going to further explore the genetic and biomarker characteristics of this non-hospitalised MHO group in a well-characterised sub-group of MDCS, the so-called Cardiovascular Cohort (MDCS-CC) [15]. Admittedly the MHO group is not without risk factors or metabolic abnormalities, and thus strictly speaking not completely metabolically healthy, but these adverse factors did not lead to severe disease causing need of hospitalisation until a mean of 56 years at MDCS baseline. Thus, it is more important to us to focus on the consequences of obesity (hospitalisation) than the levels of ever changing risk factors or prescribed drugs, many of these with infrequent use due to suboptimal compliance.

## Other Examples of Unexpected Risk Protection

Why is it of importance to find out more about a subgroup of MHO individuals with a better prognosis than expected? This is not only because we need to increase knowledge about the heterogeneity of obesity in the population, or to develop a more diverse treatment strategy based on personalised medicine to offer people with obesity, but also in order to explore if specific protective mechanisms can be found or not. This is a strategy that in the end could eventually bring new drug targets if such protective mechanisms (if any) could be mapped, or if genetic analyses could reveal loss-of-function (LoF) mutations of harmful genes, as for PCSK-9 that lead to new drug development [16], but instead linked to obesity and cardiovascular risk. Such models of “extremes” can also be found in other areas of cardiovascular medicine, for example in subjects with the Supernormal Vascular Aging (SUPERNOVA) phenotype, as recently described [17], or in patients with type 1 diabetes of long duration but escaping major complications from the cardiovascular system, the kidneys or retinopathy [18, 19].

Still, we have reason to believe that MHO subjects are not fully protected from complications. Sooner or later such cardiovascular complications will develop also in the MHO subjects defined by our new definition (non-hospitalisation for somatic disease) [13, 14••], but most likely such complications may be delayed for a shorter or longer time during the life course. This could bring benefits to the individual, but also lessen the burden on the health care system.

## Potential Mechanisms to Explain MHO

Which mechanisms could possible explain the existence of MHO or the protection from cardiometabolic complications in subjects with obesity (Table 1)? One possibility is that no protective mechanisms exist at all, but that random variation could explain most of the findings. An alternative explanation could be that genetic factors involved in the regulation of hepatic function or glucose metabolism could be of importance. One indication of this is that not all obese subjects develop non-alcohol fatty liver disease (NAFLD), as an expected and very common negative consequence of obesity and insulin resistance [20]. As chronic inflammation is another well-known characteristic of obesity, and also involved in the development of cardiometabolic complications [21], it could be hypothesised that in true MHO subjects, inflammation is downregulated. This could be due to genetic factors, less pronounced NAFLD, or eventually a more benign and diverse microbiome of the bacterial content of the gastro-intestinal tract [22]. Future studies should try to elucidate further on the role of chronic inflammation, as now a recent intervention treatment study has shown that the reduction of inflammation by use of very specific mono-clonal antibody therapy (canakinumab against Interleukin 1-beta, IL-beta) was associated with a reduction of cardiovascular risk and events in the randomised, placebo-controlled Canakinumab Anti-Inflammatory Thrombosis Outcomes Study (CANTOS) trial [23].

**Table 1:** Potential mechanisms contributing to metabolically healthy obesity (MHO), targets for further research

• Genetic variants, loss-of-function (LoF) mutations	
• Reduced chronic inflammation	
• Changes in adipose tissue composition	
• Variants of adipocyte function	
• Protection from non-alcohol fatty liver disease (NAFLD)	
• Gastro-intestinal microbiota variation	
• Less sedentary life-style (“fat and fit”)	
• Early life programming?	

### The Heart in Obesity—Unresolved Questions

The link between obesity and cardiovascular changes leading to increased CVD morbidity and mortality is well established (both myocardial infarction and ischemic stroke), mediated by metabolic and vascular pathophysiological traits as well as other mechanisms. Even though it has been shown in in some, but not all, epidemiological studies that weight reduction is associated with decreased CVD risk, so far the results of studies on the effects of weight-reducing drugs, with the aim of reducing cardiovascular morbidity and mortality, have been disappointing [24]. One potential explanation for this is that the CVD risk associated with obesity is not causally related to overweight but instead to some other factor which is increased in parallel, for example insulin resistance or chronic inflammation. So, whereas it is clear that obese patients are at increased risk of hypertension, CVD, and heart failure, identification of new drug-modifiable biological pathways causally related to both obesity and CVD still remains challenging. Recently, the GLP-1 agonists have revealed a lot of attention due to their favorable effect in reducing cardiovascular risk in high-risk diabetes patients [25, 26]. Existing on the market to date for the treatment of obesity is the GLP-1 agonist liraglutide, but other similar drugs are also being tested, for example semaglutide. Its efficacy is comparable with other available agents and greater than that seen with orlistat or lorcaserin, but slightly less than weight loss seen with phentermine/topiramate combination treatment. However, liraglutide offers the unique benefit of improved glycemic control in addition to weight loss. Although it has been shown that liraglutide, apart from lowering weight and blood glucose in subjects with diabetes at high cardiovascular risk, also reduces mortality and CVD risk [26], additional studies are needed to determine its long-term efficacy and safety profile in non-diabetic obese patients [27].

Even if drug treatment still is under development to address obesity and its complication, the role of bariatric surgery is better established for long-term benefits, also for improving cardiac structure and function [28]. Such surgical interventions could also be considered for MHO subjects to relieve from symptoms of musculoskeletal and joint pain, or for improving self-esteem and reduce psycho-social stigma.

### Less Risk of Hypertension than Expected—Why?

In the area of hypertension, there are also examples of subjects that have lower than expected blood pressure levels, for example in Iraqi immigrants to Malmö, Sweden, as compared with native, Caucasian Swedes [29]. Even if other features of the metabolic syndrome, such as increased waist circumference and dyslipidemia, were more pronounced in the Iraqis than in the Swedes, the blood pressure elevation was not in relative terms. This could be due to protective factors such as a better renal function [30], as shaped by evolutionary selection in subjects exposed to a very hot climate when the preservation of body fluids and keeping the electrolyte balance is of utmost importance. In a similar way, it was reported that Pima Indians also have somewhat lower mean blood pressure as compared with Caucasians, and also a lower sympathetic nervous activation [31], another factor that could impact on blood pressure regulation, but also on obesity when less energy is spent. Further studies are needed to disentangle biological factors from the socio-cultural factors that may apply during a migration process when lifestyle changes may occur.

## Future Research Directions

If a more benign category of obesity could be defined, this would be a step forward in a similar way that, based on a new proposal for definition of diabetes, milder forms of diabetes could be defined with a less harmful course according to risk of complications [32•].

For understanding MHO, we need both observational studies on obesity and risk in different populations and ethnicities, but also basic science when characteristics of adipocytes from MHO subjects as well as the cardiovascular phenotype, including blood pressure regulation, should be investigated. Increased knowledge of genetic traits could bring insights into protective variants, for example loss-of-function mutations of harmful variants. In addition, the study of downregulated chronic inflammation could reveal protective patterns, possible to study by use of proteomics [33]. Finally, a better understanding of the role of GI microbiota and its interaction with dietary patterns could provide new perspectives. Also the role of the “fat and fit” phenotype, with corresponding personality traits should be more studied, as this could modify the current simplified message of weight loss in all obese subjects in a uniform way. In fact, to keep weight stable in middle age without the annually expected weight increase of 0.5–1.0 kg/year, as seen in many people, could bring a new message to some overweight/obese people, i.e. that keeping yourself weight-stable and fit is better than to be lean and unfit [34]. Lessons could also be learned from the Japanese sumo wrestlers that stay fat and fit during their sports career even if the high degree of obesity will increase risk factors [35], but after their career in many cases undergo a period of de-training and de-eating to avoid or lessen obesity complications.

## Conclusions

The ongoing debate on whether MHO exists or not is important to bring new understanding and a more realistic clinical view on obesity. Most likely, the obesity phenotype is more heterogeneous than previously thought, similar to the modern view on the varying subtypes of diabetes carrying different risk of complications [32•]. If protective mechanisms associated with MHO could be identified, new treatment targets for new interventions (drugs, diet) could be defined. More direct research aims involve characterisation of adipocytes and cardiovascular function of MHO subjects, as well as the role of chronic inflammation, hepatic function, and GI microbiota to understand this condition [6••]. In the end, even if people with MHO could be identified, most likely the majority of them will neither escape cardiovascular complications nor diabetes in the long run, but if disease onset could be postponed by a decade or more, this could mean great benefits both for the individual and the health care system.
